# Transcriptomics, Proteomics and Bioinformatics in Atrial Fibrillation: A Descriptive Review

**DOI:** 10.3390/bioengineering12020149

**Published:** 2025-02-04

**Authors:** Martina Belfiori, Lisa Lazzari, Melanie Hezzell, Gianni D. Angelini, Tim Dong

**Affiliations:** 1 School of Medicine and Surgery, Università degli Studi di Milano-Bicocca, 20126 Milano, Italy; m.belfiori@campus.unimib.it (M.B.); l.lazzari13@campus.unimib.it (L.L.); 2 Bristol Veterinary School, University of Bristol, Langford House, Langford, Bristol BS40 5DU, UK; mh16511@bristol.ac.uk; 3 Bristol Heart Institute, Translational Health Sciences, University of Bristol, Bristol BS2 8HW, UK; qd18830@bristol.ac.uk

**Keywords:** bioinformatics, transcriptomics, proteomics, atrial fibrillation, machine learning

## Abstract

Atrial fibrillation (AF) is the most frequent cardiac arrhythmia, with an estimated five million cases globally. This condition increases the likelihood of developing cardiovascular complications such as thromboembolic events, with a fivefold increase in risk of both heart failure and stroke. Contemporary challenges include a better understanding AF pathophysiology and optimizing therapeutical options due to the current lack of efficacy and adverse effects of antiarrhythmic drug therapy. Hence, the identification of novel biomarkers in biological samples would greatly impact the diagnostic and therapeutic opportunities offered to AF patients. Long noncoding RNAs, micro RNAs, circular RNAs, and genes involved in heart cell differentiation are particularly relevant to understanding gene regulatory effects on AF pathophysiology. Proteomic remodeling may also play an important role in the structural, electrical, ion channel, and interactome dysfunctions associated with AF pathogenesis. Different devices for processing RNA and proteomic samples vary from RNA sequencing and microarray to a wide range of mass spectrometry techniques such as Orbitrap, Quadrupole, LC-MS, and hybrid systems. Since AF atrial tissue samples require a more invasive approach to be retrieved and analyzed, blood plasma biomarkers were also considered. A range of different sample preprocessing techniques and bioinformatic methods across studies were examined. The objective of this descriptive review is to examine the most recent developments of transcriptomics, proteomics, and bioinformatics in atrial fibrillation.

## 1. Introduction

Atrial fibrillation (AF), the most frequent cardiac arrhythmia [1], defined as the presence of rapid and multiple irregular depolarizations within the atria [2], represents a growing public health challenge.

AF is associated with increased morbidity and mortality, with an estimated five million cases globally [3], and it is a major burden and cost of health care [4,5]. It raises the likelihood of developing cardiovascular complications such as thromboembolic events and a fivefold increase in risk of both heart failure and stroke [6,7]. In 2020, AF was predicted to represent between 0.9% and 1.6% of NHS expenditure, and it is expected to increase over the next two decades.

The prevalence of AF is estimated to be around 1–2% in the general population, but it is as high as 10–17% in ≥80 years old [8,9]. Moreover, as the population is progressively growing older, the prevalence is expected to increase [2].

Aging represents the primary factor responsible for the development of this arrhythmia [10]. Other common risk factors include both noncardiovascular and cardiovascular conditions [11], with arterial hypertension, obesity, and diabetes mellitus identified as significant predisposing factors [12].

In the past 30 years, there has been an increase in understanding of this condition, supporting the management of atrial fibrillation [13]. Contemporary challenges include a better understanding of AF pathophysiology and optimizing therapeutical options due to the current lack of efficacy and adverse effects of antiarrhythmic drug therapy [14]. Nonetheless, the identification of novel biomarkers in biological samples would greatly impact the diagnostic and therapeutic opportunities offered to AF patients.

## 2. Relationship of Biomarkers with AF Pathogenesis

Markers for AF pathogenesis categorized by different modalities are presented in Table 1. Normal physiological and pathological cellular activities continuously modify genetic products, primarily mRNAs and proteins. In order to comprehend both healthy and diseased molecular mechanisms in cells, it is essential to effectively monitor the cellular or organism composition of mRNAs and proteins, known as the transcriptome and proteome, respectively. The combination of these “omics” techniques may be the secret to deciphering intricate data required to improve understanding of the AF biological system and to produce more comprehensive theories. Since blood plasma samples are less invasive to collect compared to atrial tissue samples requiring biopsy, both blood plasma and atrial tissue markers were considered separately. While some proteins, e.g., n-cadherin, are shown within the transcriptomic sections, these were reported as transcriptomics products in studies that focused on transcriptomics analysis. Few genomic-focused AF studies with datasets were found during this search. It was apparent that the current focus of research is on biomarkers found by transcriptomic and proteomic studies, which then correlate with associated genes. Hence, the focus was on these two modalities, with biomarkers commonly discussed across studies.

### 2.1. Transcriptomics

#### 2.1.1. PITX2 (Paired-like Homeodomain Transcription Factor 2)

Encoded by the *PITX2* gene [32], this protein physiologically regulates the right-left differentiation of the embryonic heart. In adults, PITX2 expression is heart-restricted, specifically to the left atrium in its PITX2c (cardiac) isoform [33]. Gene expression analyses show that *PITX2c* is involved in the expression of ion channels and desmosomal genes. Genome-wide association studies found genetic variants in the AF population, especially on chromosome 4q25, where *PITX2* can be found. Hence, modifications in *PITX2* expression and their possible link to AF are of interest [34]. Reyat et al. suggest that, according to their findings, reduced left atrial PITX2 could predispose patients to recurrent AF after AF ablation [15].

#### 2.1.2. BMP10 (Bone Morphogenetic Protein 10)

This protein is a ligand of the TGF-beta (transforming growth factor-beta) superfamily of proteins. Binding to TGF-beta receptors leads to recruitment and activation of SMAD family transcription factors regulating gene expression. It plays an important role in cardiovascular development including cardiomyocyte proliferation [35].

BMP10 in adults is mainly expressed in right atrial myocytes and repressed in left atrial tissue, regulated by *PITX2* [15,36]. Due to the difficulty of directly measuring PITX2 levels in patients, plasma BMP10 levels have been investigated as possible surrogates; in particular, elevated BMP10 levels were found to represent a reduced atrial PITX2 expression [15]. In addition, Ko et al. found downregulation of blood plasma BMPR1A (Bone morphogenetic protein receptor type-1A) in AF patients, further demonstrating the importance of the BMP signaling pathway in myocardial remodeling [25]. This family of plasmatic biomarkers requires further study in relation to the prevention of AF.

#### 2.1.3. LncRNA

Long noncoding RNAs (lncRNAs) are a subclass of noncoding RNAs (ncRNAs) exceeding 200 nucleotides in length. These molecules are gaining recognition as key regulators of cellular processes such as development, differentiation, and metabolism. Notably, lncRNAs have been recently found to be implicated in heart development; hence, their aberrant expression could be linked to various heart diseases, including atrial fibrillation (AF) [18,37]. LncRNAs also affect miRNA functions.

A study by Tang et al. [17] explored the connection between AF recurrence following catheter ablation and lncRNA-mRNA regulatory networks. Their findings suggest that post-ablation AF recurrence is associated with immune responses and myocardial fibrosis due to extracellular matrix remodeling. This is supported by many studies showing a strong relationship between immune-inflammatory responses and AF [38].

Through transcriptomics analysis, TMEM-AS1-20, a lncRNA, emerged as a critical regulator of five differentially expressed genes: FGFR1, IGF2, COL6A1, UACA, and HSPG2. Among these, FGFR1 and IGF2 have been previously confirmed to be associated with immune responses and fibrosis. Hence, TMEM51-AS1-201 appears to play a crucial role in AF recurrence after catheter ablation by modulating these processes, making it a potential target for preventing AF recurrence after ablation.

Other studies have also investigated the potential role of lncRNAs in the pathophysiology of AF. Xu et al. [39] identified two key lncRNAs with significant differential expression in AF: NONHSAT098586, which was the most upregulated, and NONHSAT040387, which was the most downregulated.

Their findings suggest that the differential expression of lncRNAs may be influenced by AF and atrial remodeling. The study further highlighted several transcription factors, including GATA1, TAF7, and EBF1, as potentially critical regulators of lncRNA expression during AF development.

#### 2.1.4. miRNAs

The transition from a healthy myocardium to a diseased state involves intricate changes in gene expression, leading to corresponding alterations in protein expression and activity. Studies have highlighted the role of microRNAs (miRNAs) in these pathological processes through posttranscriptional regulation [40]. miRNAs are short, noncoding RNA molecules, typically 20–22 nucleotides long, that bind to target sequences within the 3′ untranslated regions of genes, promoting mRNA instability or inhibiting translation. Each miRNA can regulate multiple gene products, and each gene product can be targeted by multiple miRNAs [41]. Consequently, shifts in miRNA expression patterns can significantly impact key cellular mechanisms driving cardiac pathology [4].

Interestingly, chronic AF did not have a significant impact on miRNA expression in LA tissue, whereas miRNA expression in the RA was notably affected by AF, with 47 miRNAs showing differential expression compared to the control sinus rhythm. This finding contrasts with most omics studies referenced in this paper, which primarily observed significant changes in the LA. One proposed explanation for this difference is the availability of tissue. In the study, LA appendage tissue was only removed from patients with severe dilation, whereas RA appendage tissue was removed from all patients undergoing valve surgery. Many of the miRNAs identified in this study have previously been associated with hypertrophy (miR-23a, miR-1, miR-133a), fibrosis (miR-133a, miR-30, miR-1, miR-21), or arrhythmia (miR-1, miR-133a) in the myocardium [42,43].

A recent meta-analysis by Menezes et Al. examined the sensitivity and specificity of circulating microRNAs as biomarkers for AF. They concluded that miRNA-2, miRNA-150, and miRNA-133a were consistently associated with the development of AF across several studies. Hence, using circulating miRNAs as biomarkers is a noninvasive approach with high sensitivity and specificity, which is valuable for clinical identification of AF [44].

#### 2.1.5. circRNAs

circRNAs are a large class of RNAs: they are versatile, single-stranded RNA molecules found across species, which regulate biological processes by acting as transcriptional regulators, miRNA sponges, protein templates, scaffolds, and many other functions currently under investigation. Dysregulations of circRNAs have been found to play a role in various diseases; therefore, they have the potential to serve as novel biomarkers for diagnosis, prognosis, and predicting therapeutic responses in many diseases, including AF [45,46].

In a study by Zhang et al. aimed at evaluating the sensitivity and specificity of these biomarkers for the diagnosis of new-onset AF in the postoperative setting (PoAF), hsa_circRNA_025016 was found to be upregulated in patients with new-onset AF with high diagnostic accuracy. It was concluded that it holds potential as a biomarker for the prediction of PoAF and could enable the targeting of patients who are at risk of developing this condition. Its specific mechanism is, however, still unclear, and future studies may be helpful to elucidate its role [19].

Additionally, another of the datasets considered in this paper (whose linked publication is still pending) analyzed the differences in plasmatic circRNA expression between patients with AF and control subjects in sinus rhythm. The results of sequencing show that the expression of circRNA is quite different between the two groups. Therefore, circRNA could be implicated in the occurrence and development of atrial fibrillation and may render useful biomarkers [47].

### 2.2. Proteomics

#### Muscle Proteins (Titin, Cadherin, Desmin, Myomin)

The integrity of sarcomeres and their components is essential for preserving cardiomyocyte contractility and ensuring proper electrical conductance. Here, we report the findings on how disruptions in both muscle proteins and extracellular matrix (ECM) components can act as key drivers in the initiation of AF.

### 2.3. Sarcomeric Proteins

#### 2.3.1. Titin

Titin is the largest protein in humans and plays a crucial role in the assembly and function of vertebrate striated muscles. It connects individual microfilaments within the sarcomere, helping to maintain the balance of forces between its two halves. The size and elasticity of these cross-links are crucial determinants of the extensibility properties of muscle sarcomeres, making titin’s structural integrity essential for normal myocardial function [48].

Notably, several loss-of-function variants of titin have been linked to AF, with titin-truncating variants (TTNtvs) specifically associated with early-onset AF [49,50].

**Figure 1:**
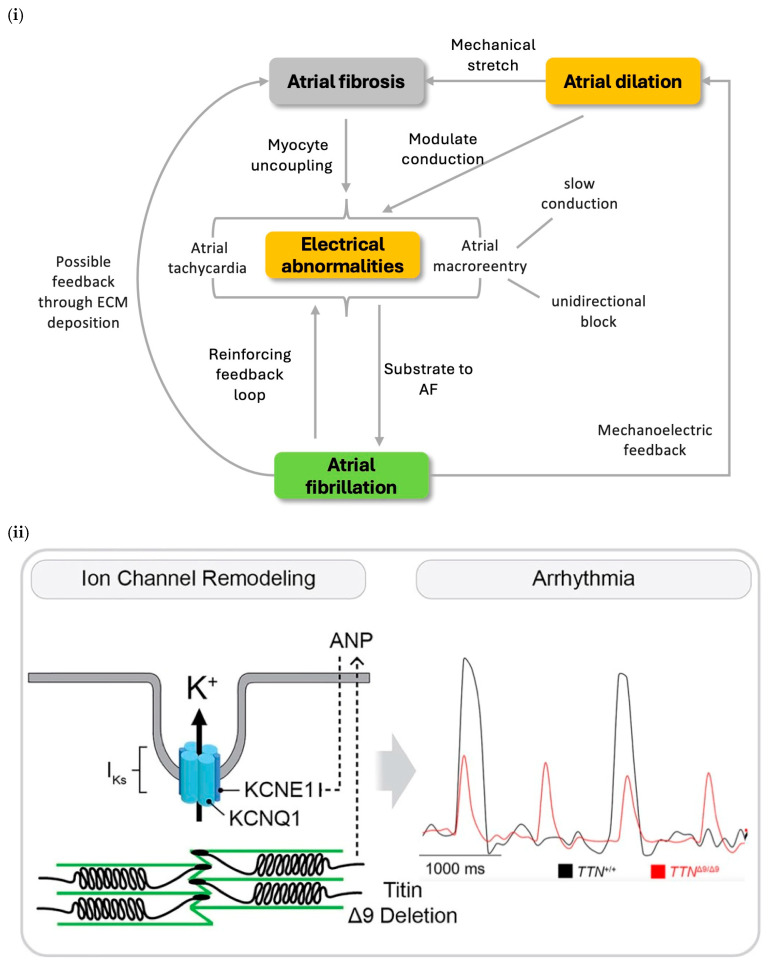

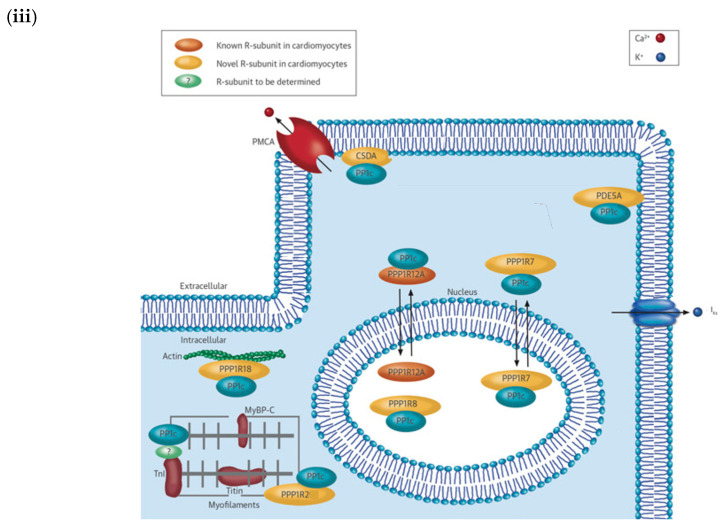
(**i**) Arrhythmogenic structural remodeling in AF development. Atrial fibrosis and dilation induces electrical abnormalities including atrial macroreentry and tachycardia, which promotes AF development; positive feedback branches are also shown; ECM—extracellular matrix. AF—atrial fibrillation [54]. (**ii**) Titin-associated ion channel remodeling. A deletion of 9 amino acids (Δ9) in the Titin protein induces an aberrant increase in atrial natriuretic peptide (ANP) expression, which in turn decreases both action potential amplitude and duration of the delayed rectifier potassium (I_Ks_) channel, leading to AF; KCNQ1 and KCNE1 are subunits of I_Ks_ channel; ${TTN}^{+/+}$: wild-type titin gene; ${TTN}^{\Delta 9/\Delta 9}$: titin gene with deletion of 9 amino acids. Taken from the article by Jiang et al. [30] with modifications. (**iii**) Protein phosphatase 1 member c (PP1c) interactome remodeling: PP1c was found to have aberrant behavior due to binding with PPP1R7, PPP1R18, CSDA—cold-shock domain protein A, PPP1R2, and PDE5A—Phosphodiesterase 5A; PPP1R12A is already known to bind; TnI—troponin I; MyBP-C—Myosin binding protein-C; PMCA—plasma membrane Ca^2+^ ATPase; taken from the article by Chiang et al. [27] with modifications.

Through proteomic analysis, Jiang et al. [30] demonstrated that deleting just nine amino acids (Δ9) from the titin A-band significantly increased the risk of AF by reducing atrial contractility and promoting ion channel remodeling (Figure 1ii), specifically through enhanced I_Ks_ activity. This altered titin variant played a dual role, contributing to ion channel-dependent remodeling and impairing atrial contractility, further highlighting its impact on atrial function and arrhythmogenesis.

Titin has also been identified to be subject to altered ubiquitination in the context of AF. In a pioneering study, Wu et al. [31] used quantitative proteomics to identify proteins with changes in ubiquitination during AF and to investigate how these specific alterations contribute to the functional changes underlying AF onset. Among the proteins exhibiting differential ubiquitination, titin stood out as having the highest number of ubiquitinated and modified sites in AF tissues, as compared to non-AF tissues.

This finding underscores once again titin’s critical role in maintaining cardiomyocyte stability during the cardiac cycle, as disruptions to its proper functioning can increase susceptibility to AF development.

#### 2.3.2. Myosin Heavy Chain 6,7 (MYH6, MYH7)

Myosin is the molecular motor protein responsible for driving muscle contraction in the heart. It consists of two heavy chains and four light chains. In the mammalian heart, two heavy chain isoforms are expressed, differing in their ATPase activity: alpha-myosin heavy chain (α-MHC), which exhibits faster ATPase activity, allows for quicker cross-bridge cycling and faster contractions, while beta-myosin heavy chain (β-MHC) has a slower ATPase activity, enabling more sustained and energy-efficient contractions [51]. In healthy humans, the slow isoform β-MHC predominates in the cardiac ventricles, whereas α-MHC is mainly expressed in the atria [52], reflecting their functional specialization.

Wu et al. [31] identified MYH6 as the second most ubiquitinated protein in AF tissue samples, following titin. The mechanisms underlying this modification remain unclear, highlighting the need for further investigation.

Additionally, MYH7 expression was found to be altered in AF tissue samples. It was found to be subject to a heightened expression in the atria of patients with chronic AF [22].

#### 2.3.3. Myomesin

Myomesin is a family of proteins located in the M-line of the sarcomere structure, where they contribute to the intricate protein network that maintains the proper alignment and interaction of the contractile filaments. Among the family members, Myomesin 1 (MYOM1) and Myomesin 2 (MYOM2) are well-established components of the M-band, where they bind tightly to titin. On the other hand, Myomesin 3 (MYOM3) has only been recently identified as part of the M-band [53].

All three myomesin isoforms—MYOM1, MYOM2, and MYOM3—are highly expressed in the heart. Recent research has linked alterations in these proteins to AF.

Wu et al. [31] identified multiple ubiquitination sites on these proteins in AF tissue samples: 14 on MYOM1, 12 on MYOM3, and 7 on MYOM2.

Additionally, MYOM1 was also reported to be upregulated in the LAA CMs as compared to the RAA in patients with chronic AF [22], highlighting the potential role these proteins might have in atrial remodeling during disease progression.

It is now evident that disruptions in key sarcomeric proteins, such as titin, myosin heavy chains, and myomesin, are closely linked to the pathogenesis of AF. As seen, altered ubiquitination and expression patterns of these proteins contribute to impaired contractility, ion channel remodeling, and atrial structural changes. However, the precise mechanisms that link these alterations to AF onset are still unclear. Hence, these findings highlight the need for further research.

### 2.4. Cadherin and Connexin

As demonstrated, structural remodeling plays a pivotal role in AF development [54], making it a critical area of study to better understand the mechanism underlying AF pathogenesis.

One of the known key features of arrhythmogenic structural remodeling is fibrosis (Figure 1i) [54,55]. However, the molecular mechanism linking AF and fibrosis remains incompletely understood. Addressing this gap, Adam et al. [20] investigated the mediators involved in fibrotic remodeling.

Their findings revealed that, in the left atrium (LA) of patients with AF, both N-cadherins and connexin-43 (Cx43) are upregulated. This process is mediated by angiotensin II-induced Rac1 activation, which stimulates connective tissue growth factor (CTGF). The activation of this signaling cascade contributes to structural remodeling by promoting interstitial fibrosis through the upregulation of N-cadherins and Cx43.

N-cadherins, which are cell adhesion proteins, are critical for maintaining cell-cell contact and regulating cytoskeletal complexes. In cardiomyocytes, N-cadherin serves as a key transmembrane component of adherens junctions, which facilitate mechanical coupling between cells. Connexins, on the other hand, are gap junction proteins that ensure proper intercellular communication, essential for maintaining coordinated depolarization in cardiac tissue. Cx43, the predominant cardiac connexin, is integral to these processes, and its altered expression or function has been linked to conduction velocity changes that predispose to arrhythmias [56].

Adherens junction formation is also essential for the proper assembly of gap junctions, as N-cadherin plays a regulatory role by transducing signals that direct the localization of Cx43. When this process becomes dysregulated, connexins are redistributed to lateral cell borders, disrupting normal intercellular ion conductivity. This disruption facilitates the onset of arrhythmias by impairing the coordinated electrical activity required for cardiac function [20,57].

### 2.5. Decorin

Deranged electrical conduction and fibrosis in AF are not only influenced by the dysregulation of muscle proteins but are also closely linked to alterations in extracellular matrix (ECM) proteins. Matricellular proteins, which regulate interactions between cells and the ECM, are particularly important indeed.

Notably, the ECM can be the driver of pathological processes, such as fibrosis and hypertrophy, under disease conditions. Studies have shown that ECM deposition within the cardiac interstitial space can facilitate the establishment of ectopic pacemakers and the development of membrane potential dysregulation by disrupting homogeneous stimulus conduction [58].

In this context, Barallobre-Barreiro et al. [29] investigated the ECM composition of atrial appendages in patients undergoing coronary artery bypass grafting (CABG), aiming to identify ECM-related differences associated with AF pathogenesis.

Their findings highlighted alterations in decorin processing, a matricellular protein, as a potential biomarker in AF.

Decorin, a member of small leucine-rich proteoglycans (SLRPs), functions not only as a structural component of the ECM but also as a mediator of cell signaling by interacting with and modulating the activity of growth factors and surface receptors [59].

First, decorin protein levels were found to be reduced in the patients developing AF postoperatively. Then, a novel decorin cleavage site, absent in patients with sinus rhythm, was observed in atrial appendages of those with AF. This cleavage site is located near the N-terminal domain of decorin, adjacent to the myostatin-binding region (Ser49-Leu50), and generates a decorin form lacking the glycosaminoglycan chain at Ser34. The resulting decorin peptide suppresses the activity of myostatin, an inhibitor of muscle growth, which promotes cardiomyocyte hypertrophy.

Decorin also physiologically interacts via the C-terminal region with CTGF, inhibiting its activity [59]. C-terminal truncation may indeed result in diminished repression of CTGF activity in human atria, promoting fibrosis. Of note, this phenomenon was mainly observed to occur in LAA, as compared to the LV.

These findings suggest that endogenous decorin cleavage products may contribute to atrial remodeling and arrhythmogenesis by modulating the local bioavailability of growth factors such as connective tissue growth factor (CTGF) and myostatin, providing a novel perspective on the molecular mechanisms underlying AF.

#### 2.5.1. AF Related Ion Channels

In general terms, the most relevant families of ion channels expressed in cardiac myocytes include sodium channels, potassium channels, calcium channels, chloride channels, and sodium/calcium exchangers. Their correct individual functioning and interaction result in the action potential of the cardiac cell, hence producing the rhythmic contraction of the heart muscle. The pathogenesis of AF is known to be related to the remodeling of some channels, in particular sodium, potassium, and calcium channels [60].

The altered function of ion channels may play an important role in AF progression, making some ion channels potential candidates for drug intervention [55]. Multiple studies have analyzed and highlighted the differential expression of ion channels in AF atrial tissue.

Franco et al., in their transcriptomic study, describe an interesting change in the pattern of expression of ion channels. While various potassium (KCNJ3, KCNJ5), calcium (CACNA1C), or sodium (SCN5A) channel genes show a decrease in expression in cardiomyocytes in AF, other components such as HCN2 (K/Na hyperpolarization-activated cyclic nucleotide-gated channel 2) or KCNH7 (Kv11.3) are increased [22]. Notably, Ayagama et al. also reported that their transcriptomic analysis identified upregulation of potassium/sodium hyperpolarization-activated cyclic nucleotide-gated channel 1 (HCN1) [9]. HCN is a cation channel activated by hyperpolarization: it contributes to the native pacemaker currents in both the heart and neurons. HCN1 protein is mostly found to be expressed in the human SAN rather than the atria, while HCN2 expression is less specific to SANthan HCN1 [61].

Yao Jiang et al., in their study, focus instead on chloride channel expression in AF patients with rheumatic valve disease due to the already reported possible role of these channels in the pathophysiology of other cardiovascular diseases. Their transcriptional analysis found differential expressions of some chloride channels, including CLICs. The subsequent proteomic study confirmed this involvement. These findings indicate that chloride channels play an important role in the pathophysiology of AF in patients with heart valvular diseases [8].

Another interesting point to investigate is the regulation of expression of ion channels or their posttranslational modifications and how they impact their activity in relation to AF. The role of protein phosphatases (PPs) and PITX2c were highlighted in the studies analyzed. *PITX2* has been discussed in another section, but as stated, it is responsible for the expression regulation of several genes related to potassium channels and calcium handling, further validating its role in AF pathogenesis [34].

Protein phosphatases (PPs) regulate the phosphorylation level of ion channels and Ca^2+^ handling proteins in the heart. Expression and activity levels of PP1 (serine/threonine protein phosphatase type 1) are shown to be increased in patients with chronic AF. Remodeling of the PP1 interactome (Figure 1iii), involving inhomogeneous changes of protein phosphorylation levels across different subcellular components (e.g., hyperphosphorylation of RyR2 and hypophosphorylation of L-type Ca^2+^ channel), could be one of the main causes of subcellular heterogeneity in protein phosphorylation associated with AF pathogenesis [27].

Further strengthening the idea that differences in calcium handling is also a transcriptomic study from Tsai et al., showing differential expression in AF samples in genes related to calcium uptake and release in the sarcoplasmic reticulum, namely RyR2, SERCA2, and IP3R1 [23].

Overall, considering the increasing presence of similar findings across various studies in the last decade, it seems reasonable to think that dysregulation of ion channels and ionic currents might be a therapeutic target to halt AF progression. We have not found evidence of plasmatic biomarkers relating to ionic channel mutations that could aid in diagnosis, but this might be worthy of further investigations.

#### 2.5.2. Miscellaneous

##### Endolysosomal Proteins

Endolysosomes (EL) are crucial for regulating intracellular trafficking, proteostasis, and calcium signaling, all of which are vital for heart function. However, their specific role in atrial fibrillation (AF) remains unclear. A study conducted by Ayagama et al. on a goat model of AF identified upregulation of the AMPK pathway and expression of EL-specific proteins, such as GAA, DYNLRB1, CLTB, SIRT3, CCT2, which were absent in whole tissue lysates. They have also observed structural abnormalities, including autophagic vacuoles, irregular mitochondria, and glycogen deposits. These findings suggest that ELs play a role in the long-term progression of AF. Further research into their role in the pathogenesis of AF could result in new potential targets for drug development and biomarker identification [9].

##### Citrate Synthase

Teng et al. conducted a study on murine models to analyze the proteome profile in angiotensin II (Ang II)-induced atrial fibrillation (AF), as hypertension and elevated Ang II are key risk factors for AF [62]. They identified several differentially expressed proteins (DEPs) in atrial tissues exposed to Ang II, primarily linked to mitochondrial oxidation-reduction processes and the tricarboxylic acid cycle.

Notably, citrate synthase was significantly downregulated in Ang II-infused atria. Overexpression of citrate synthase in cardiomyocytes was found to reduce susceptibility to AF and atrial remodeling in mice, accompanied by enhanced ATP production, upregulation of mitochondrial oxidative phosphorylation complexes I–V, and reduced oxidative stress. These findings highlight the protective role of citrate synthase in AF development, suggesting that increasing its expression could be a promising therapeutic strategy for AF [26].

##### IGFs and IGFBPs

In a study from Staerk et al. evaluating potential plasmatic biomarkers linked with AF, it was observed that lower levels of IGF1 (insulin-like growth factor 1) and increased levels of IGFBP1 (insulin-like growth factor-binding protein 1) are associated with a higher hazard of incident AF [24].

The insulin-like growth factors (IGFs) are synthesized by almost all tissues and are important mediators of cell growth, differentiation, and transformation [63]. Aging and decreasing levels of IGF1 are strongly associated [64], and likewise is the relation of aging and AF [65]. However, the exact reason for the inverse correlation between IGF1 and AF is still not well understood.

Several proteins, including IGFBP1, bind to and interact with IGF1. IGFBPs play a role in regulating the turnover, transport, and tissue availability of IGF1 [66]. Previous studies already linked some members of the IGFBPs family to AF, namely IGFBP3 and IGFBP7 [67], and now with new evidence on the link with IGFBP1 too, the relationship with these biomarkers seems ever more plausible [24].

##### BNP

Together with IGF1 and IGFBP1, NT-proBNP was also found to be strongly correlated with AF in the Staerk et al. study [24]. NT-proBNP is the inactive fragment that is released alongside BNP when the precursor molecule (proBNP) is cleaved; due to the longer half-life (90–120 min versus 20 min for BNP) and higher stability, it is sometimes used as a surrogate biomarker [68].

NT-proBNP has been well described as a biomarker to indicate ventricular remodeling and predict major cardiovascular events including AF in previous studies [69,70] and was found also in another of the papers that object to this discussion. A similarly structured analysis of possible plasmatic biomarkers of AF conducted by Ko et al. showed that NT-proBNP remained significantly associated with incident AF after statistical adjustments [25].

## 3. Druggable Targets and Compounds for AF

Finding proteins and genes linked to the pathophysiology of AF that also represent effective drug targets is an ongoing effort in research. Many modern studies focus on unveiling possible new options for prevention or cure of this condition.

Although our review focuses on presenting state-of-the-art on several biomarkers discovered to have predictive links with AF, some of these also represent druggable targets in current times: ion channels and miRNA, together with connexin, appear to be promising targets.

### 3.1. Ion Channels

A search of the Therapeutic Target Database [71] showed that many of the drugs currently under study for AF appear to target ion channels, probably due to their long-known implication in the pathophysiology of disease, as previously discussed. Currently available antiarrhythmic drugs, such as quinidine, procainamide, and disopyramide, unfortunately, have limited applications due to cardiac (pro-arrhythmic) and extracardiac side effects [72].

Recent efforts have been focusing on targeting ion channels selective to the atria, trying to reduce the risk of ventricular pro-arrhythmia of more classical AADs (antiarrhythmic drugs); nevertheless, not many channels are truly atrial selective, and moreover, the remodeling occurring during disease progression that we have previously discussed may hinder the expression of possible AAD targets [73]. Novel targets include, among others, small conductance Ca^2+^-activated K^+^ channels, which seem to be atria selective, and acetylcholine-activated inward-rectifier K^+^ channels.

### 3.2. Connexin

In a study by Bikou et al., Cx43 was linked to the pathophysiology of AF (previously discussed in relation to Adams et al. study [20] and gene therapy targeting this protein was demonstrated in vivo to hold potential for prevention of atrial arrhythmias [74].

### 3.3. MiRNA

As previously highlighted in this review, modifications in miRNA expression have been identified to have an important relationship with AF, mainly linked to the structural and electrical remodeling seen in the course of the disease [4,16]. Efforts to design therapeutical strategies based on these molecules are ongoing. In general terms, the therapeutic concept of targeting miRNAs as a therapeutic tool could be achieved by either inhibiting overexpressed miRNA using antagonisms (anti-miRNA) or mimicking the function of downregulated miRNA through miRNA mimics [75].

Some interesting studies have demonstrated that modifying the expression of specific miRNA in vivo can lead to beneficial results. Among these, miRNA-21 is of paramount importance, as its upregulation has been repeatedly shown to be linked to atrial fibrosis and atrial fibrillation.

Pradhan et al. [76] reported that anti-miR-21 treatment effectively induced anti-fibrotic processes in vitro. Similarly, Xiona Xu et al. [77] highlighted the positive impact of anti-miR-21 in reducing AF in animal models.

### 3.4. Additional Druggable Targets

Apart from the biomarkers already discussed in this review, many novel druggable targets for AF have been highlighted in several publications over the past decade.

A big retrospective cohort study recently conducted by Peng et al. [78] focused on proteomics data from many circulating proteins to identify both predictors and therapeutic targets of AF. Their findings highlighted a strong correlation between the condition in question and two specific proteins, COL4A1 (Type IV collagen) and RET (Receptor tyrosine-protein kinase). While COL4A1 is not considered druggable as it functions as a structural protein, RET appears to be a promising target instead, being currently also of interest in the development of many cancer-related therapies.

Four other promising druggable proteins for AF were uncovered by the study of Wang et al. [79] in a proteome-wide Mendelian randomization analysis: TNFSF12 (tumor necrosis factor ligand superfamily member 12), for which drugs are in clinical trial in the context of rheumatoid arthritis and tumors, IL6RA (interleukin-6 receptor subunit α), FCGR2B (low-affinity immunoglobulin γ Fc region receptor II-b), and ANXA4 (annexin A4), for which drugs have already been approved and are in use for many autoimmune conditions and cancers.

Some of these targets (ANXA4, IL6R, TNFSF12) were also corroborated by another recent work by Ning et al. [80]

Lastly, a promising compound that has been attracting attention in recent times seems to be berberine. It is a natural compound already known for its numerous beneficial cardiovascular effects employed in the management of other conditions such as myocardial ischemia and hypertension [81].

Berberine has been tested as an antiarrhythmic drug in clinical trials for many years. It was proven to have an antiarrhythmic effect in patients with chronic congestive heart [82]. Additionally, berberine was found to be effective in reducing the occurrence of POAF (postoperative atrial fibrillation) after coronary artery bypass grafting in a double-blind randomized control trial performed by Zhang et al. [83].

Proposed molecular targets of berberine include AMPK (adenosine monophosphate-activated protein kinase), PI3K (phosphatidylinositol 3-kinase), AKT (protein kinase B), and NADPH oxidase [84]. Some of these such as AMPK have already been highlighted in our review in relation to cardiac remodeling. Additional studies report that the mechanisms at the base of the antiarrhythmic effect of berberine may be linked with the suppression of delayed after-depolarization by a reduction of Na^+^ influx [85].

## 4. Sample Processing Devices and Technologies

Due to various reasons such as resource availability, market competition across commercial suppliers, and technological advancements, different sample processing technologies have been reported in the literature. Table 2 present key sample processing devices developed in order to identify the transcripts and proteins present in the AF biological system.

### 4.1. Transcriptomics

In transcriptomics studies, methodological approaches that are commonly used include RNA sequencing (RNA-Seq) techniques and microarray platforms.

**Figure 2:**
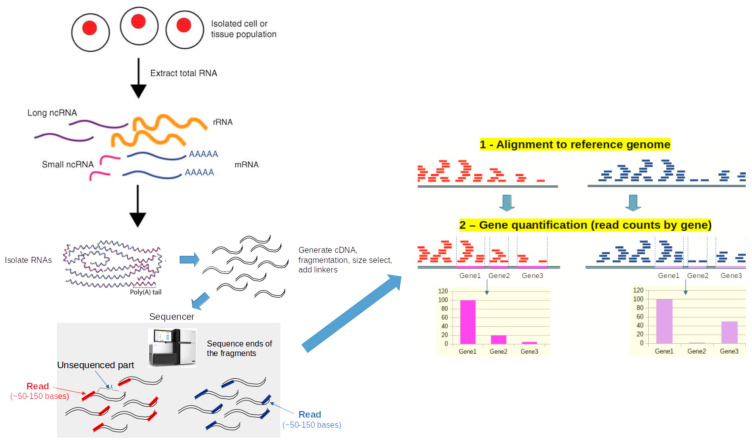
Overview of RNA sequencing. First, the desired biological material (such as cells or tissues) is used to extract RNA. Second, subsets of RNA molecules are isolated using a particular process, like ribo-depletion to eliminate ribosomal RNAs or poly-A selection to enrich for polyadenylated transcripts. After reverse transcription transforms the RNA into complementary DNA (cDNA), sequencing adaptors (linkers) are attached to the ends of the cDNA fragments. These are inputted into the sequencer for sequencing ends to generate reads after PCR amplification. The reads are matched to a reference genome to produce transcripts. The number of reads that align to each exon or full-length transcript is then counted in order to estimate the expression level of each gene. Taken from the article by Kukurba and Montgomery [86] with modifications.

RNA-Seq is a next-generation sequencing (NGS) technique that uses the capabilities of high-throughput sequencing methods to deliver a high-resolution, comprehensive view of the entire transcriptome (Figure 2) [86]. The transcriptome is highly complex, encompassing diverse coding and noncoding RNA species [87]. Earlier transcriptome analysis methods, such as hybridization-based microarray technologies and sequence-based approaches, faced several limitations, including their inability to identify novel genes. The introduction of high-throughput NGS has transformed transcriptomics, enabling RNA analysis by sequencing complementary DNA (cDNA). Compared to traditional methods, RNA-Seq offers several advantages, providing detailed, quantitative insights into gene expression, alternative splicing, and allele-specific expression. A typical RNA-Seq workflow involves isolating RNA, converting it to complementary DNA (cDNA), preparing a sequencing library, and sequencing it on an NGS platform.

Instruments in RNA-Seq include the Illumina NovaSeq 6000 and Illumina HiSeq systems. The Illumina NovaSeq 6000 is known for its high throughput, capable of generating over 20 billion paired-end reads per run, translating to more than 6000 gigabases (Gb) of data, making it ideal for large-scale studies [88]. It also offers high sensitivity and accuracy, allowing the detection of low-abundance transcripts and novel isoforms. Furthermore, despite its higher initial investment, it is cost-efficient for large projects due to its massive output, resulting in a lower cost per base compared to older platforms, and its broad flexibility facilitates its applicability to a wide range of outputs [89]. However, it requires a larger sample size, and it is subject to more stringent library requirements [90]. Concerning read lengths, NovaSeq 6000 supports read lengths up to 2 × 150 base pairs that, while sufficient for many applications it, may be limited for studies requiring longer reads [89].

The Illumina HiSeq, on the other hand, is a well-established and robust platform suitable for mid- to high-throughput needs. It balances cost and performance effectively for transcriptomic studies but is slower than the NovaSeq and has a higher cost per base [91]. However, it offers lengths up to 2 × 250 bp, providing an advantage for applications that benefit from longer reads [92].

In general, Illumina RNA-Seq techniques offer highly sensitive results and can be used for whole transcriptome sequencing, making it ideal for discovery-driven studies. The choice between the NovaSeq 6000 and HiSeq platforms should be guided by project requirements, including desired throughput, read length, budget, and available infrastructure. For large-scale projects demanding high throughput and cost efficiency, the NovaSeq 6000 is advantageous. In contrast, for applications benefiting from longer read lengths or when working within certain budgetary or spatial constraints, the HiSeq series may be more appropriate.

Microarrays are hybridization-based technologies designed to measure gene expression levels across a predefined set of transcripts. As one of the earliest approaches employed in transcriptomics studies, they offered a high-throughput solution at a relatively low cost. Despite their advantages, microarrays have several notable limitations: they require prior knowledge of the target sequences, are prone to cross-hybridization artifacts when analyzing highly similar sequences and have a limited capacity to accurately quantify genes with very low or extremely high expression levels [92].

Common instruments include the Affymetrix GeneChip and Agilent Microarrays. The Affymetrix GeneChip is one of the most widely used commercial microarray platforms, given that it can be used to monitor the expression of every gene in the genome, making it cost-effective for studies focused on known gene sets. However, unlike RNA-Seq, it is less sensitive in detecting rare transcripts and cannot identify novel transcripts, as its function is limited to the probes present on the chip [93].

Similarly, Agilent Microarrays offer high flexibility, good sensitivity and specificity for targeted studies, and affordability for large sample sizes with predefined gene sets. However, they share the same limitations as Affymetrix GeneChip, being restricted to known transcripts and being less capable of detecting isoforms or rare RNAs.

Overall, microarrays are more suitable for hypothesis-driven studies, where the aim is to analyze the expression pattern of a specific known set of genes. Their simplicity and lower costs make them more accessible and approachable than RNA-Seq, but their inability to detect novel transcripts or provide detailed information limits their application in discovery-based research.

Hence, the choice of which technique to use depends on the specific aims and resources of the study.

### 4.2. Proteomics

Mass spectrometry plays a critical role in peptide and protein analysis thanks to its speed, sensitivity, and adaptability. It facilitates the sequencing of peptides, the detection of various posttranslational modifications, and the measurement of absolute and relative protein levels. Its ability to identify and quantify thousands of proteins in complex samples makes it an invaluable tool in systems biology research [94].

A system for mass spectrometry is made of several components, namely an ionization system, a mass analyzer, and a detector. Different options exist for each component, allowing one to tailor the system based on the research needs [95].

Each system has unique strengths and is chosen based on the analytical needs, such as sensitivity, resolution, throughput, or the nature of the sample [95,96]. Differences in ionization techniques encompass electron ionization (EI), matrix-assisted laser desorption/ionization (MALDI), and electronspray ionization (ESI). Mass analyzers separate ions based on their mass-to-charge (m/z) ratio, differing based on resolution, accuracy, and speed of analysis. Detectors are instead needed to translate ion separation into quantifiable signals. These are often integrated with the corresponding mass analyzer systems. Quadrupole, Time-Of-Flight, and Orbitrap mass analyzers are commonly used in research [96]. Quadrupole analyzers are cost-effective and robust: they facilitate compound screening due to their high compound fragmentation, where ions are bombarded by neutral gas molecules such as nitrogen or argon in a process called collision-induced dissociation [97]. They selectively stabilize ions of specific m/z ratios, making them also ideal for targeted analysis and quantification of small to medium-sized molecules [98]. Time-of-flight (TOF) mass analyzers provide fast, high-resolution analysis by measuring ion travel times to determine m/z ratios, making them ideal for complex mixture analysis and structural studies in proteomics and metabolomics [99,100]. Orbitrap mass analyzers deliver exceptional resolution, mass accuracy, dynamic range, and isotope fidelity by measuring ion oscillation frequencies in an electrostatic field [101], making them ideal for high-throughput proteomics and metabolomics requiring detailed molecular analysis [96].

Mass analyzers can also be combined to perform tandem mass spectrometry (MS/MS). It can use identical analyzers in series, like TOF/TOF systems, or combine different analyzers into hybrid instruments, such as Q-TOF (Quadrupole and TOF [97]) or Q-Trap (combining a triple quadrupole to a linear ion trap [102]), for enhanced analytical flexibility. Linear iron traps [103] are mass spectrometry instruments based on the use of electric fields to confine ions in a linear chamber, allowing subsequent fragmentation and analysis; they are often times combined with a triple quadrupole mass analyzer. Quadrupole mass analyzers are instead dynamic mass filters and are reliable instruments for quantitative analysis [104].

Hybrid systems combine different performance characteristics offered by various types of analyzers into one mass spectrometer, rendering them helpful for a more comprehensive analysis [105].

The studies analyzed in this paper employed different methods, with a slight predominance of Orbitrap-based systems. Only two studies [8,31] have reported using TOF-based systems. This might be due to the higher resolution of analysis offered by Orbitrap-based systems.

Due to there being a trade-off between speed and resolution [101], different types of Orbitrap systems have been employed, and most studies actually described hybrid systems: some studies [27,29] used LTQ Orbitrap, where the Orbitrap analyzer is interfaced to a linear quadrupole ion trap (resolution power > 100,000) [106]. One of the analyzed studies [9] used Orbitrap Fusion Lumos, consisting of a tribrid system including a quadrupole mass filter, linear ion trap, and Orbitrap mass analyzers, resulting in improved speed and usability compared to a quadrupole Orbitrap (resolving power 500,000, speed of acquisition up to 30 Hz) [107]. The majority of the studies considered [2,22,26,27,28,30] used the Q-exactive system, another hybrid architecture combining a high-performance quadrupole mass filter with a high-resolution (resolving power up to 140,000), accurate-mass Orbitrap resulting in good performance, speed (max 12 Hz) and overall versatility [108].

Most of the studies under discussion have coupled MS analysis with liquid chromatography. LC consists of molecule separation based on differential retention time between a mobile phase and a stationary phase. Liquid Chromatography with tandem mass spectrometry (LC-MS/MS) is a powerful analytical technique that combines the separating power of liquid chromatography with the highly sensitive and selective mass analysis capability of MS [102]. This technique enhances analyte separation from matrix components, thereby improving sensitivity and precision. Based on the particle size desired, high-performance LC and ultra-high-performance LC are also available [109].

Some of the studies under discussion [8,62] highlight the application of the iTRAQ (isobaric tags for relative and absolute quantification) [110] technique in proteomics. This method involves covalently labeling the N-terminus and side-chain amines of peptides in a protein digest with isobaric tags [111]. These mass-balanced tags are indistinguishable during initial mass spectrometry but become separable during the subsequent step, enabling simultaneous analysis of multiple samples by using distinct iTRAQ reagents for each. This multiplexing capability significantly enhances throughput and reduces experimental variability. During MS/MS, fragmentation releases reporter ions with unique masses, facilitating the relative quantification of peptides and their corresponding protein [110].

In conclusion, the most employed method in the analyzed studies appears to be liquid chromatography coupled with tandem mass spectrometry (LC-MS/MS), utilizing a hybrid mass analyzer based on Orbitrap technology. This approach is widely adopted in proteomics research due to its ability to deliver accurate, reliable results and its suitability for the detailed analysis required in the studies under consideration.

## 5. Statistical Transformations and Tests Applied for Datasets

Statistical analysis is indispensable for transforming raw data into meaningful biological insights, validating hypotheses, and communicating findings effectively to the scientific community.

The reviewed studies [2,4,9,15,17,19,20,21,22,23,24,25,26,27,29,30,31,39] employed a diverse range of statistical methods to analyze transcriptomic and proteomic data in atrial fibrillation (AF), tailored to address the specificity of the datasets.

Dimensionality reduction techniques [9,22] are increasingly popular in exploratory biomedicine science, as big databases are evermore widespread: they aim to reduce the dimensionality of these datasets, improving interpretability without losing much information [112]. Examples of these techniques include principal component analysis (PCA), a statistical method used to approximate the original dataset using only a few “principal components”, which are a few linear combinations of the original variables, maximally explaining the variance of all the variables [113]. PCA, used by Ayagama et al. [9], was applied to the analysis of sample distribution and to the identification of key components driving differences between experimental conditions in group separation (AF vs. control). Multiple co-inertia analysis is another technique used for integrative analysis of high dimensional omics datasets; it can assess relationships based on covariance across features of multiple datasets by projecting their eigenvectors to a common scale [114]. Multiple co-inertia analysis was applied in the Alvarez et al. study [22], enabling the integration and analysis of multimodal datasets like transcriptomics and proteomics.

Comparative statistical tests were also found to be central to these analyses. Parametric tests like paired *t*-tests, used to analyze samples from the same population, or unpaired *t*-tests, also called two-sample *t*-tests or Student’s *t*-tests, for samples coming from different populations [115], were widely used [4,19,20,27,29,30,39] for those variables meeting the necessary criteria (normally distributed, equal variance and continuous) [116]. Their purpose is to assess mean differences between two groups, and they are generally chosen for their simplicity and efficiency under the abovementioned assumptions [117]. Normality of data distribution is commonly assessed via some tests such as Shapiro–Wilk [2,15,26], used for smaller datasets, or Kolmogorov–Smirnov [15], used when a larger sample size (n ≥ 50) is analyzed [118]. In case of failure to demonstrate normal data distribution, nonparametric alternative tests were employed, like the Mann–Whitney *U* test [2,15,20,26,30] and the Kruskal–Wallis [26] test were employed. Nonparametric analysis focuses on the order of the data size rather than on the value of the data per se, and although this can result in a loss of information on the original data, this analysis has more statistical power than the parametric test when the data do not satisfy the normality assumption [119].

For datasets involving multiple comparisons, where simple Student’s t-tests would not be suitable, ANOVA (with one categorical independent variable) and two-way ANOVA (with two categorical independent variables) [120] were implemented to evaluate interactions among multiple factors, often rendering more useful in the analysis of multivariate omics datasets. These tests assess the relative size of variance among group means compared to the average variance within groups [121]. If detecting differences in variance, post-hoc comparison procedures such as Bonferroni correction and Newman-Keuls are then commonly used to determine which group means differ after ANOVA testing [122].

To address the challenges of false positives in large-scale omics datasets, false discovery rate (FDR) corrections (e.g., the Benjamini–Hochberg method [123]) and Bonferroni adjustments [124] were consistently applied [2,19,23,24,25,30]. These corrections ensured the reliability of significant findings by mitigating the effect of type I error rates (false positive rate) [125].

Multivariable regression models, such as Cox proportional hazards and logistic regression, aim to describe the behavior of a response variable in terms of other explanatory variables [126] and were used to examine the relationship between biomarkers and AF outcomes, accounting for covariates and interactions [15,19,24,25].

Overall, these methods are able to handle the multifaceted nature of omics datasets, improve interpretability, and increase statistical rigor. However, challenges such as small sample sizes, data heterogeneity, and reliance on specific distributional assumptions highlight areas for further methodological development.

## 6. Bioinformatic Packages, Tools or Methods

Bioinformatics methods are pivotal for extracting meaningful insights from complex biological data. The reviewed articles employed several widely used computational tools and databases, particularly for functional enrichment, differential expression analysis, and protein interaction studies. A high-level summary of key approaches used across studies is provided in Table 3.

Functional enrichment analyses were a common approach across multiple studies. Gene Ontology (GO) [17,22,31] and Kyoto Encyclopedia of Genes and Genomes (KEGG) databases [17,26,31,39] were consistently used to annotate genes and identify overrepresented biological processes and pathways. Visualization of enrichment results was often performed using R packages, collections of functions, data, and documentation to analyze and interpret complex biological data.

Differential expression analysis of RNA-Seq data frequently utilized tools like DESeq2 and Limma, both of which were applied in multiple studies to identify genes with significant expression changes [15,21,26].

DESeq2 is a statistical tool designed for analyzing RNA-Seq and other high-throughput sequencing data to identify differentially expressed genes or transcripts between experimental conditions [127]. Limma (Linear Models for Microarray Data) is a statistical package primarily used for analyzing gene expression data, including RNA-Seq and microarray data. It employs linear models to identify differentially expressed genes across multiple experimental conditions [128].

These tools incorporated methods such as empirical Bayes or the Benjamini–Hochberg procedure for multiple test corrections to ensure statistical robustness and to control the false discovery rate (FDR).

**Figure 3:**
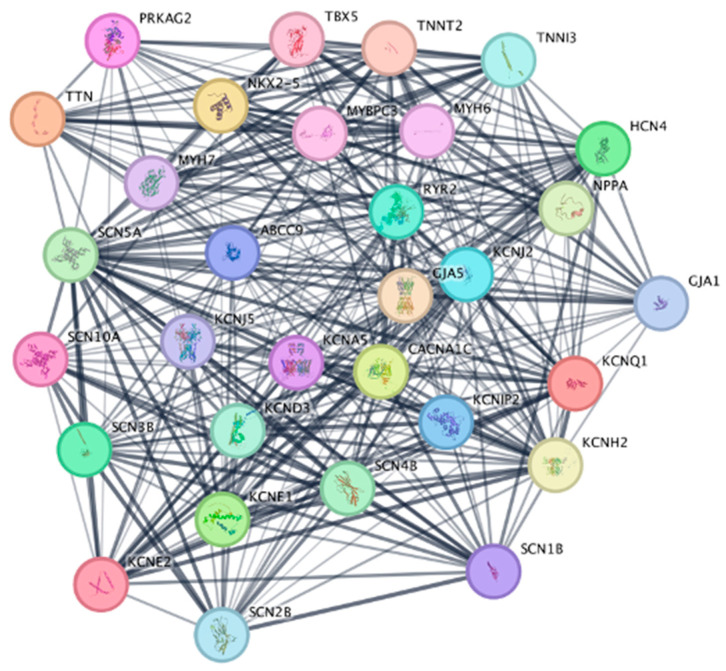
STRING analysis for Atrial fibrillation [17]. Cytoscape with MCODE analysis displaying the highest score module for Atrial fibrillation with the resulting protein set presented through STRING at a threshold of 0.4 confidence cut-off; TTN—titin; HCN, SCN, and KCN ion channels are displayed for example; newer experimental proteins such as DCN—Decorin are not identified as expected.

Protein–protein interaction (PPI) analysis was another recurring technique performed using the STRING database (Figure 3) to identify enriched interaction networks. STRING is a biological database of known and predicted protein interactions from various sources, including scientific text, experimental literature, and computational predictions. The interactions include direct (physical) and indirect (functional) associations. A confidence score is determined for each protein interaction once the data has been weighted and integrated. All predicted or imported interactions are benchmarked against reference standards set by the Kyoto Encyclopedia of Genes and Genomes (KEGG). Cytoscape version 3.10.2, an open-source software platform for visualizing molecular interaction networks, along with the CytoHubba plugin, was employed to construct these networks and pinpoint hub genes critical to the studied biological processes [17,31,39].

Hierarchical clustering and pathway enrichment tools, including Metascape and DAVID, were also applied. These methods facilitated the identification of gene sets with shared functional characteristics across different experimental conditions [21].

## 7. Conclusions and Future Perspective

This review underlines the pivotal role of multiomics approaches in the discovering of novel biomarkers and mechanisms underlying AF. Transcriptomics and proteomics are clearly valuable in identifying gene-regulated pathways, ion channel remodeling, and extracellular matrix alterations as critical drivers of AF. Biomarkers uncovered through these approaches, such as PITX2, BMP10, and NT-proBNP, among others, offer potential not only for diagnostic precision but also for prognostic evaluation and therapeutic targeting.

Future research should, therefore, focus on validating these biomarkers in diverse populations and integrating multiomics datasets for a holistic understanding of AF pathogenesis. Advancements in bioinformatics may further enhance the predictive value of identified biomarkers and aid the discovery of new therapeutic targets, which are of the outmost importance for the improvement of patients’ prevention and treatment. The recent emergence of new sequencing methods, such as nanopore sequencing technology, should be investigated in terms of its applicability in the discovery of therapeutic targets or biomarkers [129]. The approach is able to sequence individual nucleotides by measuring the electric current across a polymer membrane as a motor protein drives either RNA or DNA strands through a nano-sized pore in a stepwise manner.

From a technological perspective, future studies should consider the interoperability of input data and quality control of data using bioinformatic tools. Phylotools is a useful R package that can be used to read miRNA FASTA (.fq) datasets. Smrnaseq and DOGMA are useful sets of software packages for preprocessing and quality control of FASTA miRNA datasets. Studies should also consider various feature representation methods [130]. For example, MathFeature, a feature coding approach that uses various mathematical theories to map transcriptomic or proteomic datasets into features, could be readily used for machine learning [131]. Machine and deep learning approaches should also be explored to map the relationships across the transcriptome and the proteome. For instance, iDeep is a deep learning-based framework for detecting RNA–protein interaction sites that have been proposed. The CLIP is a useful dataset for studying RNA–protein binding interactions and has been analyzed using data integration approaches such as iONMF to improve the accuracy of binding site predictions [132]. These different tools may improve our understanding of the complexity of arrhythmia and help to improve diagnostic and therapeutic options offered to AF patients.

In the future, the diagnoses of AF using advanced modeling perhaps is of lower importance since, most of the time, clinicians have knowledge of whether a patient suffers from AF through electrophysiology and clinical observations from an applicative point of view (while it is not trivial in a mechanistic perspective). What needs to be known is: which biomarker(s) is (are) more promising as targets for a pharmacological intervention? One possible strategy to find these targets is through Cytoscape by identifying the biomarkers with a higher centrality on the interaction network and/or considering the ‘reachability’ of the target(s) using pharmacological interventions, taking into consideration their location in the network module. Other machine and deep learning approaches that can take into consideration the structural relationships and distance across networks can also be considered.

## Figures and Tables

**Table 1:** Known/suspected biomarkers associated with AF pathogenesis.

Category	Tissue Type	Biomarkers	References
***Transcriptomics***	*Blood plasma*	*BMP10*	[15]
		*miR-146a-3p*, *miR-125b-5p*, *miR-34a-5p*, *miR-150-3p*	[16]
		*lncRNA: TMEM51-AS1-201*	[17]
		*lncRNA: NONHSAT040387*, *NONHSAT098586*	[18]
		*hsa_circRNA_025016*	[19]
	*Atrial tissue*	*HCN1*, *AGL*	[9]
		*Rac1*, *RohA*, *CTGF*, *N-cadherin*, *Cx43*	[20]
		*miR-21*, *miR-146b-5p*, *miR-133a&b*, *miR-30 family*, *miR-10b*, *miR100*	[4]
		*CLIC 1-6*	[8]
		*PITX2*, *BMP10*	[15]
		*ATRNL1*, *KCNN3*	[21]
		*PITX2*, *KCNJ3*,*5*, *CACNA1C*, *SCNA5*, *TTN*, *MYOM1*	[22]
		*RyR2*, *SERCA2*, *IP3R1*	[23]
***Proteomics***	*Blood plasma*	*IGF1*, *IGFBP1*, *NT-proBNP*	[24]
		*ADAMTS13*, *N-terminal pro-B-type natriuretic peptide*, *BMPR1A*	[25]
	*Atrial tissue*	*GAA*, *Rab7a*, *CTBL*, *VPS25*, *CCT2 (endolysosomal complex)*	[9]
		*CLIC 1*,*4*,*5*, *collagen type IV*	[8]
		*LCN2*, *MPO*, *MYH10*	[2]
		*OXPHOS complex*, *citrate synthase*	[26]
		*PP1c interactome: PPP1R7*, *CSDA*, *PDE5A*	[27]
		*GSS*, *Decorin*	[28,29]
		*TTNΔ9/Δ9 (deletion titin)*	[30]
		*TTN*, *MYH6*	[31]

**Table 2:** Sample processing devices.

Omics	Tissue Type	Device	Study Reference
Transcriptomics	Atrial tissue	RNA-sequence-based: - Illumina NovaSeq - Illumina HiSeq	[8,21,22]
		Microarray Platforms: - Affymetrix GeneChip - Agilent Microarrays	[4,20,23]
	Blood plasma	RNA-sequence-based: - Illumina NovaSeq	[17]
		Microarray platforms: - Agilent Microarray	[39]
Proteomics	Atrial tissue	Orbitrap-based systems	[2,9,22,26,27,28,29,30]
		TOF-based systems	[8,31]

**Table 3:** Statistical and bioinformatic methods.

**Statistical analysis techniques**	Dimensionality reduction techniques	Principal component analysis (PCA)	[9]
		Multiple co-inertia analysis	[22]
	Parametric tests	Paired *t*-test	[4,19,23,30]
		Unpaired *t*-test (Student’s *t*-test)	[19,23,27,29,30]
	Nonparametric tests	Mann–Whitney *U* test	[2,15,20,26,30]
		Kruskal–Wallis test	[26]
	Normality tests	Shapiro–Wilk test	[2,15,26]
		Kolmogorov–Smirnov test	[15]
	Multivariate analysis	One and two-way ANOVA tests	[4,8,23,30]
	Post-hoc analysis	Bonferroni correction	[23,25,30]
		Benjamini–Hochberg method	[2,19,24]
	Regression models	Cox proportional hazard	[15,24,25]
		Logistic regression	[15,19,24]
**Bioinformatics packages, tools, and methods**	Functional enrichment analysis	Gene ontology	[17,22,31]
		KEGG	[17,26,31,39]
	Gene expression analysis	Limma	[21,26]
		DESeq2	[15,21]
	Network analysis	Cytoscape	[17,39]
		Cytohubba	[17]
	Functional annotation and pathway analysis	Metascape	[21]
		DAVID	[17]
